# Temporal distribution of genetically homogenous ‘free-living’ *Hematodinium* sp. in a Delmarva coastal ecosystem

**DOI:** 10.1186/2046-9063-8-16

**Published:** 2012-07-24

**Authors:** Joseph S Pitula, Whitney D Dyson, Habibul B Bakht, Ihuoma Njoku, Feng Chen

**Affiliations:** 1 Department of Natural Sciences, University of Maryland Eastern Shore, Princess Anne, MD, 21853, USA; 2 University of Maryland Center for Environmental Sciences@the Institution of Marine and Environmental Technology, Baltimore, MD, 21202, USA

**Keywords:** Hematodinium, Life cycle, Environment, Population

## Abstract

**Background:**

Significant damage to crustacean fisheries worldwide has been associated with *Hematodinium sp.* It has been postulated that *Hematodinium* sp. requires passage through the water column and/or intermediate hosts to complete its life cycle. Thus, an understanding of the prevalence and seasonality of *Hematodinium* sp. within environmentally-derived samples should yield insight into potential modes of disease transmission, and how these relate to infection cycles in hosts.

**Results:**

We conducted a two year survey, from 2010–2011, in which 48 of 546 (8.8%) of environmental samples from the Maryland and Virginia coastal bays were positive for *Hematodinium* sp. between April and November, as based upon endpoint PCR analysis specific to blue crab isolates. Detection in both water and sediment was roughly equivalent, and there were no obvious seasonal patterns. However, there was a high detection in April water samples, which was unanticipated owing to the fact that crabs infected with *Hematodinium* sp. have not been observed in this early month of the seasonal disease cycle. Focusing on three sites of high prevalence (Sinnickson, VA; Tom’s Cove, VA; and Newport Bay, MD) *Hematodinium* sp. population diversity was analyzed using standard cloning methods. Of 131 clones, 109 (83.2%) were identical, 19 displayed a single nucleotide substitution, and 4 contain two nucleotide substitutions.

**Conclusions:**

Our data suggests a continuous presence of *Hematodinium* sp. in both water and sediment of a combined Maryland and Virginia coastal bay ecosystem. The detection of *Hematodinium* sp. in the water column in April is an earlier manifestation of the parasite than predicted, pointing to an as yet unknown stage in its development prior to infection. That the population is relatively homogenous ranging from April to November, at three distinct sites, supports a hypothesis that one species of *Hematodinium* is responsible for infections within the ecosystem.

## Background

The blue crab (*Callinectes sapidus*) fishery is of critical importance to the economics of the Chesapeake Bay region. In the United States over a third of all blue crabs come from this fishery [1]. In 2010 approximately 92 million pounds were harvested from the Chesapeake Bay and its tributaries, representing the largest amount since 1994 [2]. Blue crab populations have historically experienced regular population fluctuations, with a recent surge attributed to improved stock management practices. In the context of efforts to sustain a vigorous fishery, it is critical to monitor disease-causing agents such as the dinoflagellate parasite *Hematodinium* sp.

Worldwide, significant damage to crustacean fisheries has been associated with *Hematodinium sp.* as observed in Alaska Tanner and snow crabs (*Chionoecetes* spp.), and the Norway lobster (*Nephrops norvegicus*) from European waters [3-5]. Recognition of the broad ecological range of this parasite has led to increasing reports of infection in various fisheries [6-8]. In many affected crustacean species disease manifests as shell discoloration and ‘chalky’ hemolymph, discouraging human consumption [3]. In blue crabs the disease prevalence has been reported to be as high as 90% in Maryland and Delaware coastal bays [9], and thus the biological impact on crab survival and reproduction is likely to be significant.

An important question to be resolved is how crustaceans acquire the disease. A preliminary transmission study supported a hypothesis that wild blue crabs acquire the parasite through cannibalism and/or predation on other infected prey. In this study 11 naïve crabs were fed five grams of *Hematodinium*-infected crabmeat, and six became infected through this route [10]. However, this has been contradicted by a more recent study using a similar approach. Extremely low transmission rates were observed, and it was concluded that crabs that developed disease were most likely harboring low-level infections prior to the experiment [11]. Concurrent with these studies is a growing body of evidence that *Hematodinium* sp. may be present within marine ecosystems as short-lived dinospores [12,13], and also associated with potential zooplankton vectors, such as amphipods and crab larvae [14-16]. It is thus likely that, in nature, *Hematadinium* sp. requires passage through the water column and/or intermediate hosts to complete its life cycle [12,17].

Among diverse crustaceans, disease prevalence occurs on a seasonal basis. For the blue crab, peak infections occur between late summer to autumn [9,18]. By contrast, six crustacean species in the Clyde Sea of Scotland displayed two peaks of infection during the year. The highest peak typically occurred between February to April, with a smaller peak for several species in November [19-21]. An understanding of the prevalence and seasonality of *Hematodinium* sp. within environmentally-derived samples should yield insight into potential modes of disease transmission to various hosts.

We report on the detection of blue crab-specific *Hematodinium* sp. in the water column and sediment from 18 sampling sites within Maryland and Virginia coastal waters. The temporal distribution and genetic diversity of *Hematodinium* sp. in these samples was analyzed by cloning and sequencing methods. Our results suggest a persistent presence of *Hematodinium* sp. between April and November, with a relatively homogenous population structure.

## Results

### *Hematodinium* in water and sediment

**Figure 1:**
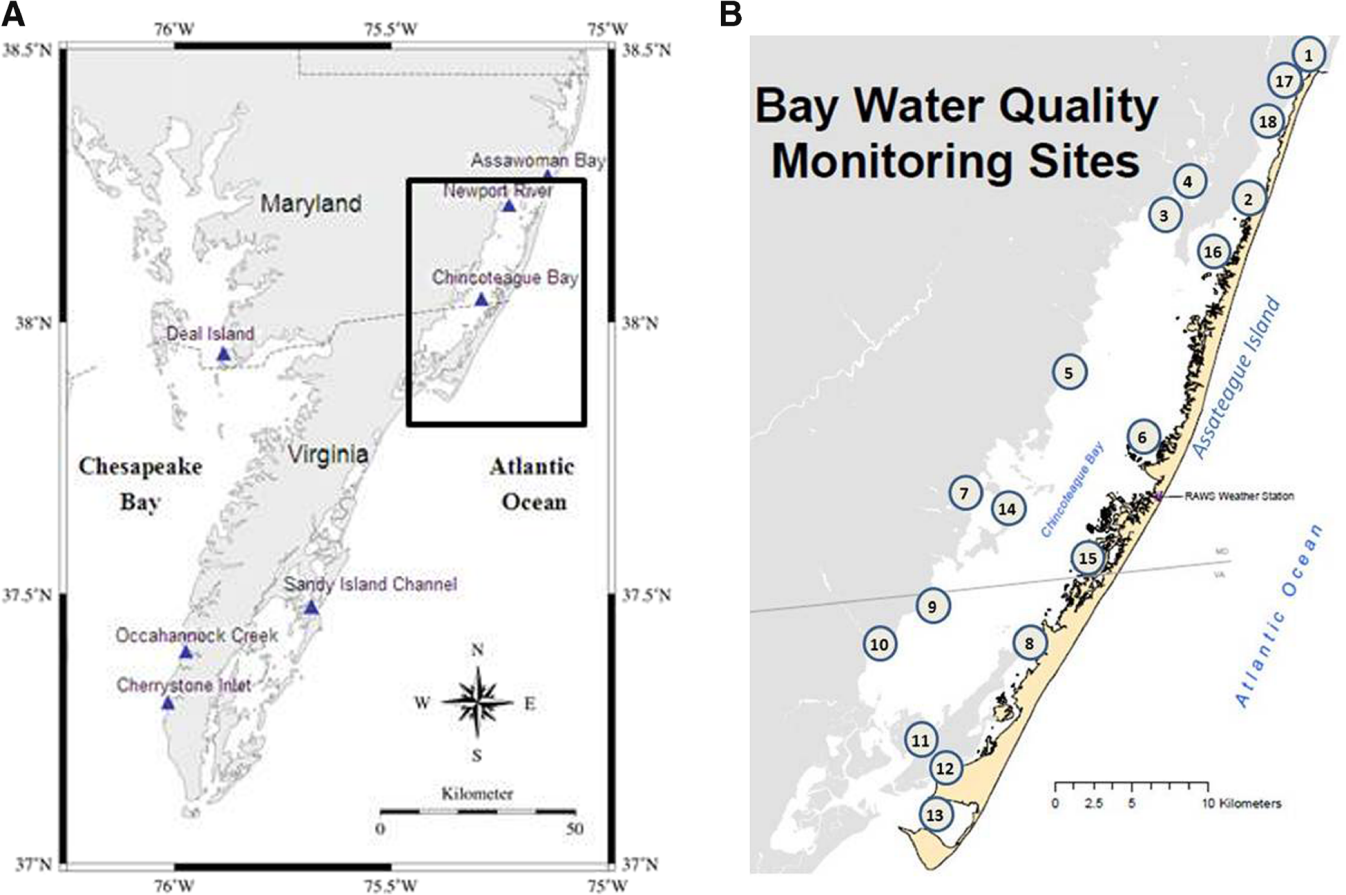
**A. Map of the Delmarva peninsula, showing both Chesapeake Bay and coastal bays.** Boxed in the inset is the study site described in this work, and is shown in greater detail in Figure 1B. B. Water quality monitoring stations in a Maryland and Virginia coastal bay ecosystem. From April through November of 2010 and 2011, sediment and water samples were collected from 18 sites south of the Ocean City inlet, and adjacent to the Assateague Island National Seashore Park. The sampling stations are monitored yearly by the National Park Service water quality program. DNA was extracted from these samples for subsequent PCR analysis to monitor the presence of *Hematodinium* sp. Figure courtesy of the National Park Service. Sites: 1, Commercial Harbor; 2, Verrazano Bridge; 3, Newport Bay; 4, Trappe Creek; 5, Public Landing; 6, Whittington Point; 7, Taylor’s Landing; 8, Wildcat Point; 9, Greenbackville; 10, Sinnickson; 11, Chincoteague Channel; 12, Assateague Channel; 13, Tom’s Cove; 14, Johnson’s Bay; 15, Cedar Island; 16, South Point; 17, Ocean City Inlet; 18, Snug Harbor.

Two seasonal surveys were conducted for the environmental presence of *Hematodinium* sp. Eighteen sites within Maryland and Virginia coastal bays were investigated, encompassing the Sinnepuxent, Newport, and Chincoteague Bays, south of the Ocean City, MD inlet (Figure 1). The time frame for sampling was initiated with emergence of blue crabs from winter hibernation in April 2010, through late summer to early autumn, and into winter hibernation beginning in November 2010. This sampling regimen was repeated in 2011 and permitted for investigation of known *Hematodinium*-positive sites during periods prior to, and throughout, a seasonal pattern of high prevalence in summer and low to non-existent infections during the winter. Sediment and plankton samples were collected from each site, and the DNA from the biota present was extracted for further analysis (see Materials and Methods).

Endpoint PCR-based analysis, using *Hematodinium*-specific primers, detected 48 of 546 sample sites (8.8%) as positive, based upon observation of a definitive band corresponding to the predicted amplicon length of 285 bp (data not shown). The distribution was relatively even throughout all months, and also between plankton and sediment samples, although positive detections for 2010 were higher than in 2011 (Table 1). In addition, *Hematodinium* sp. was never detected in water samples in May or November. Varying environmental parameters, such as dissolved oxygen and water temperature differences, did not correlate to any patterns in detection (data not shown). Three widely dispersed locations (sites 12, 16, and 17) were never positive in our assays.

**Table 1:** ***Hematodinium*sp. presence in a coastal bay ecosystem**

		**Water collection dates**	**Sediment collection dates**
Site 1	Commercial Harbor	7_10	8_11
Site 2	Verrazano Bridge	9_10	4_10; 5_10; 7_10; 8_11
Site 3	Newport Bay	6_10; 9_10	6_10; 8_11
Site 4	Trappe Creek	4/10; 7/10	8_11
Site 5	Public Landing	6_10; 9_10	5_10; 8_11
Site 6	Whittington Point		8_10; 8_11
Site 7	Taylor's Landing	4/10; 6/10; 7/10	
Site 8	Wildcat Point		8_10; 8_11
Site 9	Greenbackville	6_10	8_11
Site 10	Sinnickson	4_10; 6_10; 7_10; 8_10; 10_10	5_10; 7_10; 8_10; 10_10; 11_10
Site 11	Chincoteague Channel	7_10	
Site 13	Tom's Cove	6_11	8_10; 10_10; 8_11
Site 14	Johnson's Bay	4_10; 6_10	
Site 15	Cedar Island	7_10	
Site 18	Snug Harbor	10_10	4_10; 6_10

Shown are the sites and dates in which *Hematodinium* sp. detection occurred, based upon gene-specific PCR analysis. The sites correspond to those labeled in Figure 1B. Dates are indicated by the month, followed by the year. Typically sampling was conducted in the second week of each month, unless weather conditions precluded collection to the third week.

### *Hematodinium* and other dinoflagellates in the water column

Collectively, our data suggested that putative free-living *Hematodinium* sp. maintain a continuous environmental presence in water and/or sediment in the Delmarva coastal ecosystem. During 2010 Sinnickson, VA (site 10) was positive in our PCR screen for all months in either water or sediment, and in addition it was one of four sites in which water samples were positive in April (Table 1). The April water data was of particular interest as during this month crab infections have never been reported, and thus release of *Hematodinium* sp. cells from diseased crabs was unlikely. As Sinnickson represented a potential hotspot for environmental transmission, we sought to further explore the dinoflagellate population structure from water samples at this site.

To detect *Hematodinium* sp., along with other dinoflagellates, we generated an 18 S rRNA clone library that targeted a conserved region of the gene. We reasoned that enumeration of *Hematodinium* sp. within the library would yield a rough estimate of its abundance relative to other free-living dinoflagellate species, in addition to confirming our temporal observations. The seasons in which various dinoflagellate species are known to bloom has been established in the related ecosystem of Chesapeake Bay [22], and thus their presence in specific months provided an internal control for dinoflagellate population structure analysis.

**Figure 2:**
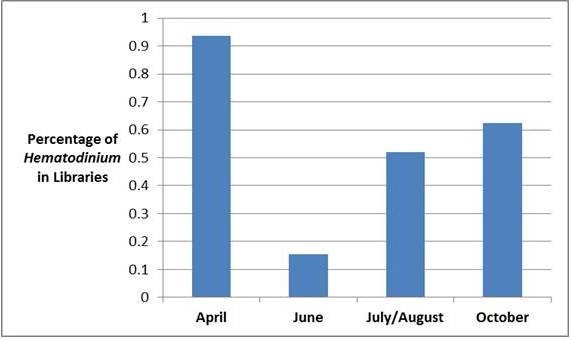
**Proportion of*Hematodinium*sp. in water samples from Sinnickson, VA in 2010.** Shown is a graphical representation of the percentage of *Hematodinium* sp. clones, relative to other species detected, as derived from clone library results presented in Table 2.

Dinoflagellate 18 S rRNA clone library results for Sinnickson in 2010 are presented in Table 2 and Figure 2, subdivided into categories of early spring (April), late spring (June), summer (July/August), and autumn (October). In total, *Hematodinium* sp. was detected in 40/70 (57%) of water samples. This percentage was unexpectedly high, and may represent some unknown sampling bias. The plankton filter used has a pore size of 20 μm which in theory should allow released amoeboid trophonts, in addition to individual micro- and macrodinospores, to pass though the net [17], but would still retain cells either in the process of division or in clumps. Alternatively, cells may associate with small plankton or particulates. Surprisingly, analysis of individual months revealed that April has the highest relative prevalence (15/16 or 94%) for *Hematodinium* sp. (see Figure 2). The temporal distribution of other dinoflagellates was consistent with the succession pattern from Chesapeake Bay [22]. *Heterocapsa rotundata*, commonly present in Chesapeake Bay through winter and spring, represented 77% (10/13) of the clones from June sequences. In summer and early autumn a mix of species that included *H. rotundata* and *Gymnodinium* sp. was observed, although the majority of dinoflagellates remained *Hematodinium* sp. (23/41 or 56%).

**Table 2:** Dinoflagellate species present in water samples from Sinnickson, VA in 2010

***Hematodinium***	**Other species**	
April	15/16	1 unidentified nanoflagellate
June	2/13	10 *Heterocapsa rotundata, 1 Peridinium sp.*
July/August	13/25	3 *Gymnodinium sanguineum, 1* Gymnodinium *sp., 3 H. rotundata* and 5 unidentified eukaryotic clones.
October	10/16	2 *Pentapharsodinium tyrrhenicum*, 1 *G. simplex, 1 Gymnodinium sp.,*
		*1 H. rotundata* and 1 *Dinophyceae* sp.

Shown are sequencing results from analysis of libraries generated using dinoflagellate-specific 18 S rRNA.

**Figure 3:**
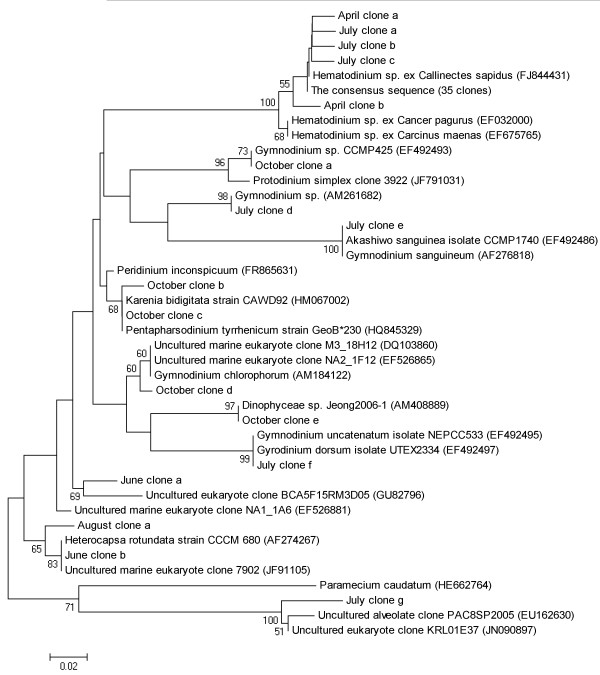
**Phylogenetic analysis based on the partial 18 S rRNA gene sequences retrieved from clone libraries.** The un-rooted Neighbor-joining tree was constructed based on the aligned DNA sequences with bootstrap value of 100, with bootstrap values less than 50 not shown.

A phylogenetic analysis of the 2010 Sinnickson samples was also performed (Figure 3). Of the 40 *Hematodinium* sp. clones in the library, 35 were identical and are labeled in the phylogenetic tree as “The consensus sequence.” Five other *Hematodinium* sp. clones, from the months indicated, contained single nucleotide polymorphisms, with the exception of April clone b which had two (data not shown). The *Hematodinium* sequences detected in the Sinnickson samples were more closely related to an isolate from *Callinectes sapidus* than to *Hematodinium* spp. isolated from other non-portunid host crustacean species such as *Cancer pagurus* and *Carcinus maenas*. Other representative dinoflagellate clones from the 2010 Sinnickson library are also included, and they are related to a diverse group of dinoflagellates (Figure 3).

### Sequence analysis of *Hematodinium* clones

It has been suggested that the species of *Hematodinium* infecting crabs in the waters of the Delmarva Peninsula is a host generalist, based on the high sequence identity of the ITS-1 region from various infected crustaceans [16]. This observation is consistent with similar data derived from analysis of both clade A and clade B *Hematodinium* disease systems [23]. We were thus interested to analyze the population structure in our study, particularly as the temporal distribution patterns suggests a continuous environmental presence at hotspot sites such as at Sinnickson, VA in 2010. In addition to Sinnickson, we concurrently analyzed Tom’s Cove, VA and Newport Bay, MD (sites 10, 13, and 3 respectively). These locations produced 37.5% (18/48) of the total positive identifications, and also represent distributions that are in the northwest, southwest, and southeast ends of Chincoteague Bay (see Figure 1B).

Clone libraries were generated from PCR products amplified by the *Hematodinium*-specific ITS1/5.8 S rRNA primers used in our initial detection assays. In Table 3 is a comparison of the relative identity of our clones derived from three hotspots of environmental presence. The populations were homogeneous, with approximately 83.2% of all clones identical (109/131). Of the remaining 22, 18 contained single nucleotide polymorphisms (SNPs). Surprisingly, these SNPS did not predominantly cluster in the ITS-1 region of the amplicon, as 10 of the 18 clones contained substitutions in the 5.8 S rRNA gene.

**Table 3:** **Relative identity of*Hematodinium*clones derived from three hotspots of environmental presence**

	**Identical sequence**	**Percentage**
**Sinnickson**		
p/w April 2010	13/16	81%
Sed August 2010	3/3	100%
Sed November 2010	25/27	93%
**Tom's Cove**		
p/w June 2010	12/14	86%
Sed August 2010	16/20	80%
Sed November 2010	25/36	69%
**Newport Bay**		
p/w June 2011	5/5	100%
Sed June 2010	10/12	83%
**Total**	109/131	83%

Clone libraries were generated from *Hematodinium*-specific ITS1/5.8 S rRNA primers used in our initial detection assays, with locations and dates of analysis. Identical sequences were present in 83% of the clones in the libraries. p/w: plankton-water samples; Sed: sediment samples.

**Figure 4:**
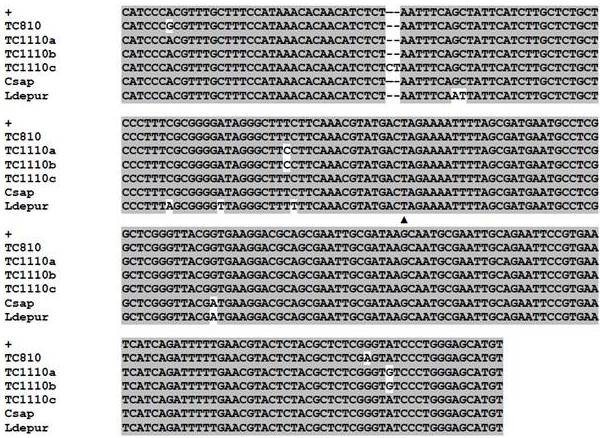
**Sequence alignment of*Hematodinium*sp. clones.** The consensus sequence from 131 environmental clones, derived from 109 identical sequences, is designated by “+”, and is aligned with the four most divergent clones in the library; one clone from Tom’s Cove (TC) in August, and three from November. Also shown is alignment with GenBank sequences from *C. sapidus* isolates (Csap: Accession DQ925229 joined with JN641990) and the portunid crab *Liocarcinus depurator* (Ldepur: Accession EF153729 joined with JN641974). The triangle (▲) indicates the boundary between ITS1 and the 5.8 S rRNA gene.

An alignment of the dominant clones in our libraries is shown in Figure 4 contrasted to sequence from a *Hematodinium* sp. isolated from an infected blue crab. A SNP between these is located at position 40 of the 5.8 S rRNA, with G substituting for A. However, it should be noted that a single clone in our library matched identically with these alternative sequences. Also shown in the alignment are the four clones containing two substitutions. Despite these minor differences, all samples were >98.5% identical or higher in sequence, suggesting a genetically homogenous population that is likely derived from a single species.

## Discussion

The *in vitro* life cycle of *Hematodinium* species has been characterized by culturing methods [17,24]. These, along with other studies [3,13], have supported a hypothesis for the mode of disease transmission in nature to entail infective dinospores, as a small portion of cultured dinospores develop into filamentous trophonts [17]. As the filamentous trophont is routinely observed in infected crustaceans from field studies [4,8,11], it is possible that released dinospores from these hosts may develop into trophonts in the water column and/or an intermediate vector. Alternatively, the dinospore may itself be the infectious stage, or may require a preparatory cyst phase. In order to explore these life cycle questions in a coastal bay ecosystem, it was important to first search for the chronological manifestations of *Hematodinium* sp. within the study site.

Based on a preliminary PCR-based screen, *Hematodinium* sp. was equally distributed in both water and sediment samples collected in 2010 and 2011 (Table 1). In most cases we detected parasite DNA in only water or sediment, but not both, for positive sites. The only exceptions were at Newport Bay, MD in June of 2010, and Sinnickson, VA in July, August, and October of 2010.

That *Hematodinium* sp. was detectable in sediment during all months was expected, as we anticipated that both free-living forms, in addition to parasites from degraded crab tissue, would be present. By contrast it was predicted that the preponderance of detections in water samples would be between June-November, as these months have traditionally been those in which blue crabs have their highest prevalence and intensity of infection [16,18], and are thus most likely to be releasing dinospores. Surprisingly, 17.4% (4/23) of our water column detections occurred in April 2010 when little or no dinospores were anticipated to be present in the water column. To our knowledge this is the earliest environmental identification of *Hematodinium* sp. in this ecosystem. Previous work in a Virginia coastal ecosystem detected *Hematodinium* sp. in the water column, but in the month of November 2007 [13]. It should be noted that a study conducted in a Georgia estuary system in 1999 and 2000 also tested for *Hematodinium* sp. in the environment prior to disease in blue crabs. It was not detectable in surface waters in March or April, but was detectable in May when blue crabs began to manifest disease [12].

To further investigate the temporal manifestation of *Hematodinium* sp. in water we analyzed samples acquired from Sinnickson, VA (site 10), which in 2010 was a ‘hotspot” of environmental detection. Using primers targeting the 18 S rRNA of dinoflagellates, the presence of *Hematodinium* sp. was confirmed within the ecological context of other resident species (Table 2). Although not strictly quantitative, the distribution of *Hematodinium* sp. clones in these months was intriguing, particularly its relative abundance in April. This month yielded 15/16 (94%) of sequences that matched *Hematodinium* sp., and coincides with the peak abundance of 20–40 mm carapace width juveniles (MDDNR personal communication,). It is also known that, in this ecosystem, juveniles have the highest disease prevalence [18]. Thus the presence of *Hematodinium* sp. in the water column at this stage may point to an important means of disease acquisition, as it is known with *Chionoecetes opilio* that actively molting crabs acquire infection [25]. It should be noted that the sampling method used in this study was capable of harvesting free-living *Hematodinium* sp., potentially in association with zooplankton. It has been suggested that macrozooplankton, such as amphipods or crustacean larvae, may harbor parasites [16,21]. In April, amphipods as vectors are a reasonable supposition. However, blue crab larval vectors during this month are unlikely, as release of larvae from females does not typically occur until May in this ecosystem.

The vast majority of *Hematodinium* sp. infections in the Chesapeake Bay region occur in the predominant crustacean species, *C. sapidus*, which has been classified as a clade A host species [26]. A recent study from Delmarva Peninsula waters has suggested that a single species is responsible for all infections [16]. We thus examined the population structure of *Hematodinium* sp. from sites that showed a high environmental presence, to determine if these reservoirs maintained one genetically homogenous species or other potential sub-species. All 131 clones from our libraries were >98.5% identical, with only four clones containing two nucleotide substitutions (Figure 4). Based on this particular ribosomal marker, our results suggest that the diversity of *Hematodinium* sp. in the Maryland Coastal Bays is low.

The consensus ITS-1 sequence in our clones is identical to ITS-1 sequences recently reported for five xalternate host species from Delmarva waters (Accession #: JN368194, JN368172, JN368154, JN368162, and JN368158) which are the: skeleton shrimp (*Caprella geometrica*), atlantic mud crab (*Panopeus herbstii*), longnosed spider rab (*Libnia dubia*), depressed mud crab *Eurypanopeus depressus*, and flat-clawed hermit crab (*Pagurus pollicaris*), respectively. In addition it is identical with the *C. sapidus* ITS-1 from an isolate in 2006 (Accession: DQ925229). Our data thus supports the previously suggested hypothesis that a single species of *Hematodinium* is responsible for infections in the Delmarva ecosystem [16]. ITS sequences in ribosomal genes are predicted to show the greatest variation as they are removed during ribosomal processing. Since the ITS1 consensus sequence we observed is identical to those in alternative hosts, and has not diverged significantly since 2006, two additional implications can be drawn. A) The *Hematodinium* sp. that infects blue crabs is not likely to have recently received new pathogen species into this ecosystem, and B) the time that it spends associated with alternative hosts is likely brief, since more variations would be predicted if its infectivity was limited to individual host species.

## Conclusions

The near continuous detection of *Hematodinium* sp. at Sinnickson, VA, and indeed within this coastal ecosystem as a whole, suggests a dynamic interplay between the host and the environment. It has been posited that *Hematodinium* sp. parasitizes primarily blue crabs, cycling through various reservoir hosts either through incidental infections or by ingestion [16]. Our data indicates that a source for these potential modes of environmental transmission and/or life cycle stages will be continuous. It should be emphasized that the work presented here is not strictly quantitative, and thus the utilization of real time PCR methodologies [13] should yield more detailed insights into the life history of *Hematodinium* sp. in an ecological setting.

## Methods

### Collection of samples

From April to November of 2010 and 2011, monthly sediment and water samples were collected from 18 sites between the northern end of the Maryland coastal bays and the southern end of the Virginia coastal bays (Figure 1). The only exception was September of 2010, where only sites 2, 3, and 5 were sampled. Typically sampling was accomplished in the second week of each month, over a two-day time span, unless weather conditions delayed collection to the third week.

For sediment collection, a Ponar grab was used to collect ~ 40 g of sediment, with two replicates per site. To collect phytoplankton samples, a plankton net with a 30 cm diameter was trawled through sub-surface water for 3 minutes at boat speeds ranging from 3–4 mph. The total water volume sampled was thus estimated to be approximately 25 m^3^ for each collection. The retention cup was fitted with a 20 μm filter to capture phytoplankton and microzooplankton. A final volume of between 30 to 50 ml of concentrated plankton was obtained in each trawl. Samples were frozen overnight at 20°C, and thawed at room temperature without shaking (to permit for settling of larger particulate matter prior to DNA isolation).

### DNA isolation from water and sediment samples

Isolation of DNA from sediment and water samples was accomplished by using the UltraClean Soil DNA Isolation Kit (MoBio Laboratories) and the Illustra Tissue and Cells Genomic Prep Kit (GE Healthcare), respectively, according to the manufacturers’ protocols. One gram of sediment was used per isolation, and DNA was re-suspended in a final volume of 100 μl of water. For DNA isolation from water samples, 200 μl was used per preparation. After DNA isolation, the samples were re-suspended in water to a final volume of 100 μl.

### PCR and cloning methods

Environmental PCR detection assays used the following primers: forward *Hematodinium* primer: (5’-CGCCTACCACTGAACTCCTC-3’); reverse *Hematodinium* primer: (5’-TGAACAGACGCTGAGACCAG-3’). Primer design was based upon *Hematodinium* sp. sequence derived from a blue crab infection (Eric Schott, personal communication, Accession # JQ815886). The forward primer anneals to a region within the 3’ end of ITS-1, and was predicted to hybridize only to clade A *Hematodinium* species [26], as the ITS-1 of clade B sequences available at the time we began our study showed significant divergence at this site. The reverse primer hybridizes to the junction between 5.8 S rRNA and ITS-2. One μl was used in each reaction, and PCR parameters were set at 58°C and 45 s for annealing, extension at 72  C for 30s, with amplification for 35 cycles. Electrophoresis of PCR products in 1% agarose gels was followed by visualization with UV light after staining with ethidium bromide. For sediment samples, a positive score for detection was given for amplification in either of the two replicates.

For cloning purposes amplicons were purified from gels using the Gene Clean Kit (MP Biomedicals). Isolated products were cloned using the TOPO TA system (Invitrogen), and were sequenced at the University of Maryland Center for Marine and Environmental Sciences at the Institute of Marine and Environmental Technology (UMCES@IMET).

For PCR reactions designed to detect dinoflagellate species, the following primers from Oldach *et al.*, 2000 [27] were used: universal dinoflagellate SSU forward primer (5’-CGATTGAGTGATCCGGTGAATAA-3’);universal eukaryotic SSU reverse primer (5’-TGATCCTTCTGCAGGTTCACCTAC-3’). Reaction conditions were set at 58°C and 45 s for annealing, extension at 72°C for 30 s, with amplification for 35 cycles. Products were identified, excised from gels, and cloned as described above.

### Phylogenetic analysis

The partial 18 S rRNA gene sequences obtained from the clone libraries were carefully checked for chimeric artifacts using the BLASTN program (http://www.ncbi.nlm.nih.gov/BLAST) and chimeric sequences were excluded from the phylogenetic analysis. Sequence alignment and phylogenetic reconstruction were performed using MEGA 5.05 software [28].

## Competing interests

The authors declare that they have no competing interests.

## Authors’ contributions

WED collected samples in 2010 and 2011, isolated DNA from sediment and water, and performed PCR identification assays. HB performed dinoflagellate population analysis. IN and JSP generated *Hematodinium* sp. population libraries. JSP and FC conceived the study and analyzed data. JSP drafted the manuscript. All authors read and approved the final manuscript.
